# Non-Destructive Quality Evaluation of 80 Tomato Varieties Using Vis-NIR Spectroscopy

**DOI:** 10.3390/foods12101990

**Published:** 2023-05-14

**Authors:** Lilija Duckena, Reinis Alksnis, Ieva Erdberga, Ina Alsina, Laila Dubova, Mara Duma

**Affiliations:** 1 Faculty of Agriculture, Institute of Soil and Plant Science, Latvia University of Life Sciences and Technologies, 2 Liela Street, LV-3001 Jelgava, Latvia; laila.dubova@lbtu.lv (L.D.); ieva.erdberga@lbtu.lv (I.E.); ina.alsina@lbtu.lv (I.A.); 2 Department of Mathematics, Faculty of Information Technologies, Latvia University of Life Sciences and Technologies, 2 Liela Street, LV-3001 Jelgava, Latvia; ralksnis@llu.lv; 3 Department of Chemistry, Faculty of Food Technology, Latvia University of Life Sciences and Technologies, 2 Liela Street, LV-3001 Jelgava, Latvia; mara.duma@lbtu.lv

**Keywords:** *Solanum lycopersicum* L., Vis-NIR spectroscopy, partial least squares regression (PLS), phytochemicals

## Abstract

Traditional biochemical methods are resource- and time-consuming; therefore, there is a need for cost-effective alternatives. A spectral analysis is one of the non-destructive techniques that are more widely used for fruit quality determination; however, references are needed for traditional methods. In this study, visible and near-infrared (Vis-NIR) spectroscopy was used to analyze the internal quality attributes of tomatoes. For the first time, 80 varieties with large differences in fruit size, shape, color, and internal structure were used for an analysis. The aim of this study was to develop models suitable to predict a taste index, as well as the content of lycopene, flavonoids, β-carotene, total phenols, and dry matter of intact tomatoes based on Vis-NIR reflectance spectra. The content of phytochemicals was determined in 80 varieties of tomatoes. A total of 140 Vis-NIR reflectance spectra were obtained using the portable spectroradiometer RS-3500 (Spectral Evolution Inc.). Partial least squares regression (PLS) and multiple scatter correction (MSC) were used to develop calibration models. Our results indicated that PLS models with good prediction accuracies were obtained. The present study showed the high capability of Vis-NIR spectroscopy to determine the content of lycopene and dry matter of intact tomatoes with a determination coefficient of 0.90 for both parameters. A regression fit of $R^{2}\;$= 0.86, $R^{2}\;$= 0.84, $R^{2}\;$= 0.82, and $R^{2}=\;0$.73 was also achieved for the taste index, flavonoids, β-carotene, and total phenols, respectively.

## 1. Introduction

The tomato (*Solanum lycopersicum* L.) is an economically important vegetable crop. Its global production quantity has increased 87% in the past six decades, from 32.4 million tons in 1961 to 251.7 million tons in 2020. China, India, Turkey, the United States of America, Egypt, and Italy are leading global tomato producers [1]. The fresh tomato and tomato products are an essential source of antioxidants, and consumption of them is characterized by various health benefits, especially reduced risk of chronic diseases [2,3,4,5].

The tomato is a climacteric fruit that differs in size, shape, color, and physical and biochemical traits [6]. Breeding for high yield, long shelf life, and good external fruit quality has impacted fruit sensory quality attributes. Therefore, during the last three decades, taste and nutritional quality have received more attention in tomato breeding programs [7]. Various factors affect consumers’ preferences and perceptions, including tomato fruit size and color, and most importantly, internal sensory quality attributes and the health value of varieties [8,9].

Nutritional and health-beneficial components of a tomato fruit traditionally have been determined by destructive, time-consuming, and expensive biochemical methods that use toxic solvents. Moreover, a small number of fruits are examined using destructive testing methods. Therefore, there is a need for quick and non-destructive alternatives that could provide a large-scale analysis [10,11].

Different imaging techniques can be used in fruit quality assessment, more specifically, Raman imaging, fluorescence imaging, magnetic resonance imaging, a soft X-ray, thermal imaging, infrared thermography, laser backscattering imaging, microwave imaging, and hyperspectral imaging. Among these, nuclear magnetic base techniques have high equipment prices; however, infrared base techniques are unsuitable for a large surface analysis, and a precise interpretation of results is difficult in the case of unpredictable temperatures. Moreover, permittivity and conductivity base techniques have slow detection speeds [12]. 

Therefore, spectral base methods combined with mathematical techniques are more dominant in fruit and vegetable quality control and prediction due to non-destructiveness and high-speed detection [13,14]. Vis-NIR spectroscopy is one of the techniques that effectively predicts multiple quality parameters in a single scan, providing a resource- and time-saving analysis [15]. This technology has been demonstrated to characterize internal quality attributes in various fruits, including apples [16], pears [17], kiwifruit [18], bananas [19], melons [20], and tomatoes [14]. Moreover, Vis-NIR spectroscopy allows for following the biochemical changes of the fruits during the ripening process, harvesting fruits at the optimum time, and assessing postharvest ripeness of climacteric fruits cost-effectively [21,22]. 

Clément et al. [23] reported simultaneously measuring tomatoes’ quality parameters in a non-destructive manner using Vis-NIR reflectance spectroscopy and chemometrics. Results showed a well-predicted tomato color index. Borba et al. [24] used a portable NIR spectrometer (Neospectra-Module, Si-Ware Systems, Egypt) based on a micro–electro–mechanical system spectrometer at a wavelength range of 1295–2611 nm. They reported the simultaneous quantification of various quality parameters in Roma, round, grape, and cherry tomatoes. Good prediction models were obtained for soluble solids, fructose, glucose, citric acid, and ascorbic acid content, and the coefficient of determination ($R^{2}$) was higher than 0.80 for all compounds. Excellent prediction accuracy of $R^{2}$ = 0.94 was obtained for titratable acidity. Moreover, the high prediction accuracy of an independent prediction set measured in the field was obtained, indicating the good robustness of obtained models. 

Although Vis-NIR spectroscopy has been widely used to determine fruit quality parameters, the research has been mainly focused on one or few cultivars, which have a small variation in color and size [25,26,27]. In our study, 80 varieties of tomato were used for the first time with large differences in fruit size, shape, color, and internal structure, which has not been previously reported. 

The aim of this study was to develop models suitable to predict six internal quality traits (i.e., a taste index, and the content of lycopene, flavonoids, β-carotene, total phenols, and dry matter) of intact tomatoes based on visible and near-infrared reflectance spectra.

## 2. Materials and Methods

### 2.1. Sampling

Tomato fruits were harvested from 2018 to 2022 in different seasons from plants grown in plastic film, polycarbonate, and glass greenhouses with or without additional lighting, including store-bought fruits. Tomatoes were tested at the full ripeness stage. 

Measurements were done with 80 varieties of tomatoes:

- red fruit varieties ‘Amaneta F1’, ‘Aurea F1’, ‘Bellastar F1’, ‘Berberana F1’, ‘Conchita F1’, ‘Elegance F1’, ‘Gardener’s Delight F1’, ’Gourmandia F1’, ‘Lancelot F1’, ’Pozano F1’, ‘Sunstream F1’, ‘Bolstar Gimli F1’, ‘Encore F1’, ‘Delizia F1’, ‘Belfast F1’, ‘Nectar F1’, ‘Cristal F1’, ‘Forticia F1’, ‘Nectar F1’, ‘Borsalina F1’, ‘Sandoline F1’, ‘Panekra F1’, ‘Sakura F1’, ‘Cocktail Crush F1’, ‘De Barao Krasny’, ‘Hector F1’, ‘Strabena’, ‘Cornue des Andes F1’, and ‘Brooklyn F1’,
- pink ‘Cipars F1’, ’Dimerosa F1’, ‘DRK936 F1’, ’Fuji Pink F1’, ‘Rosa Star F1’, ‘Rosetta F1’, ‘Pink Wonder F1’, ‘Pink Oxheart’, ‘Rhianna F1’, ‘Kongo F1’, ‘Gusto Pink F1’, ‘Pink Rock F1’, ‘Honey Moon F1’, and ‘Cassarosa F1’,
- orange—‘Apressa F1’, ’Beorange F1’, ‘Organza F1’, ‘Santorange F1’, ’Orange Wellington F1’, ‘Taijo F1’, ‘Orange Star F1’, ‘Kinkanstar F1’, and ‘Orange Queen’,
- yellow—’Bolzano F1’, ’Gualdino F1’, ‘Citrusovij Sad’, ‘Taiko’, and ‘Lemon Tree’,
- light green—‘Green Envy F1’ and ‘Limetto F1’,
- multicolored—‘Ananas’, ’Gargamel’, ‘Green Tiger’, ‘Pink Tiger’, ‘Blush Tiger’, and ‘Zebrino F1’,
- brown—‘Black Cherry F1’, ‘Bucanero F1’, ‘Chocostar F1’, ‘Chocomate F1’, ‘De Barao Chornij’, ‘Noire De Crimee’, and ‘Sacher F1’,
- and others (‘Ruskije Kolokola’, ‘Timenta’, ‘Sunstream’, ‘Toila’, ‘Pinedo’, ‘Luciestar’, ‘Delikates Moskvi’, and ‘Indigo Rose’) were included.

The average tomato mass varied from 8.6 g (‘Bellastar F1’) to 212.0 g (‘Pink Wonder F1’).

### 2.2. Non-Destructive Methods and Reference Analysis

Reflectance spectra of tomato fruits were obtained with a portable spectroradiometer RS-3500 (Spectral Evolution Inc., Haverhill, MA, USA). The RS-3500 Remote Sensing Bundle includes a fiber optic field portable remote sensing spectroradiometer, a spectral range of 350–2500 nm, and a resolution of 1 nm. The spectroradiometer RS-3500 was tuned and calibrated with a built-in white light reference before data acquisition. A total of 12–16 reflectance spectra for each variety were obtained. Spectra were represented as averages for each variety. After recording the spectra, the same fruits were used immediately for the reference measurement of quality parameters. 

The extraction and determination of phytochemicals were performed as reported in the study by Alsina et al. [28]. The following internal quality attributes were determined (Table 1): the content of lycopene (expressed as mg 100 g^−1^), β-carotene (mg 100 g^−1^), total phenols (gallic acid equivalent (GAE) per 100 g of fresh tomato mass), total flavonoids (amount of quercetin equivalents (QE) per 100 g of fresh tomato mass), dry matter (mg g^−1^), soluble solids (°Brix), titratable acidity (mg of citric acid per 100 g of fresh tomato weight), and a taste index [29].

### 2.3. Mathematical Methods and Data Analysis

In total, 158 tomato samples were used in this study. To obtain models that could predict the internal attributes listed above, partial least squares regression (PLS) was implemented, which is a common approach in the related literature [32,33]. It is applied when the number of predictors is larger than observations, as was the case in this study since there were 2250 predictors— a reflectance percentage for each wavelength. The goal of PLS regression is to compress the main information from all predictors into some relatively small amount of PLS components that could be used for the prediction [34]. The approach is similar to principal component analysis (PCA); however, this algorithm searches for components that would be correlated as much as possible with the dependent variable. 

**Figure 1:**
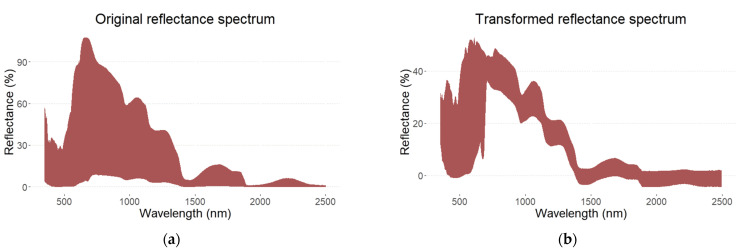
Original (**a**) and multiplicative scatter corrected and smoothed (**b**) visible and near-infrared (Vis-NIR) reflectance spectrum of intact tomato at a wavelength range of 350–2500 nm.

Before model training, pre-processing transformations were applied to improve the prediction performance. Some common methods, such as centering and smoothing as well as taking derivatives of the reflectance spectra, were investigated. Finally, the optimal performance was reached when multiple scatter correction (MSC) was applied, which reduced the light scatter that was present in the obtained spectrum data [35]. Afterwards, the Savitzky–Golay filter with third-order polynomials and window size 5 was used for smoothing, thus improving the model performance even further. Original and smoothed spectra are shown in Figure 1.

After the pre-processing stage, models were trained on 70% of the samples using k-fold cross-validation with 10 folds repeated five times. As hyperparameters, the number of PLS components was searched in the range from 1 to 40. The best-performing model was chosen based on the root mean squared error (RMSE), which is calculated as the square root of the mean of the sum of squared differences between predicted and measured values. Finally, the obtained model was validated on the hold-out sample of 30% of tomatoes. The coefficient of determination $R^{2}$ was reported for calibration and validation sets as well as the root mean squared error (RMSE), mean absolute error (MAE), and ratio of the standard error of prediction to standard deviation (RPD). All reported analyses were conducted using the software R (version 4.2.2; R Core Team, 2022) on Windows 10 × 64 (build 19045). More specifically, the package prospectr (version 0.2.6; Stevens A, Ramirez-Lopez L, 2022) (version 0.2.4) was used in the pre-processing stage and caret (version 6.0.93; Kuhn M, 2022) was used to train and validate the PLS models.

## 3. Results

### 3.1. Lycopene

The measured level of lycopene content in the sample was 3.56 ± 2.34 (sample mean ± sample standard deviation) mg 100 g^−1^ with a total range from 0 to 8.38 mg 100 g^−1^. The sample distribution was somewhat skewed and bimodal (skewness was −0.06 and kurtosis 2.03) with no apparent outliers.

**Figure 2:**
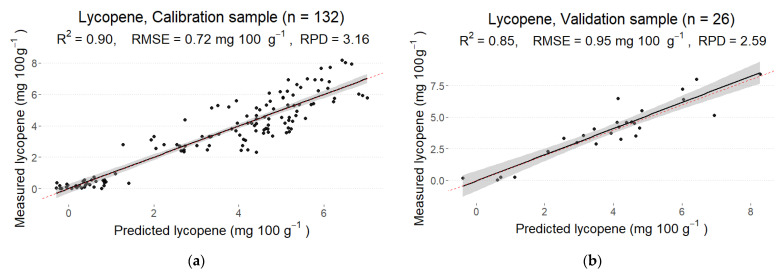
Predicted and measured values of lycopene in the calibration (**a**) and validation (**b**) sets derived from the best partial least squares regression (PLS) model from the reflectance spectrum with MSC pre-processing.

The final calibration model with the root mean squared error (RMSE) of 0.72 mg 100 g^−1^ showed a good fit with $R^{2}$ = 0.90. When applied to a hold-out validation set, $R^{2}$ = 0.85 was obtained with the RMSE of 0.95 mg 100 $g^{-1}$. The ratio of the standard error of prediction to standard deviation (RPD) for the calibration sample was 3.16, indicating a good prediction accuracy, while RPD for the validation sample was 2.59. Hence, although the model performed worse on the new samples, its predictions were still satisfactory (Figure 2). The optimal number of PLS components for the model was 10, which is relatively small compared to other models in this study.

### 3.2. β-Carotene

The level of measured content of β-carotene in the sample was 5.68 ± 2.60 mg 100 g^−1^, and it varied in the range from 0.04 mg 100 g^−1^ to 11.73 mg 100 g^−1^. The sample distribution was negatively skewed and bimodal (sample skewness was −0.18 and kurtosis 2.68). 

**Figure 3:**
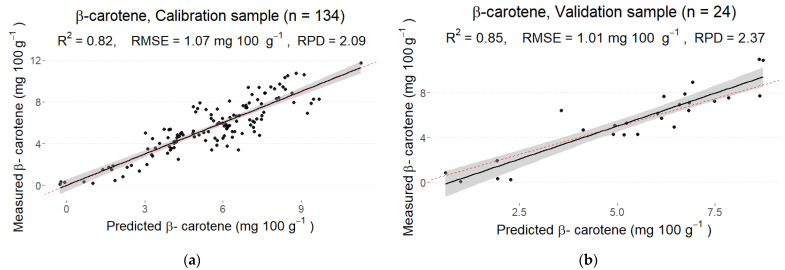
Predicted and measured values of β-carotene in the calibration (**a**) and validation (**b**) sets derived from the best partial least squares regression (PLS) model from the reflectance spectrum with MSC pre-processing.

The final calibration model with a root mean squared error of RMSE = 1.07 mg 100 g^−1^ showed a good linear fit ($R^{2}$ = 0.82). The coefficient of determination for the validation sample was $R^{2}$ = 0.85 with RMSE = 1.01 mg 100 g^−1^. Thus, the obtained model performed equally well for calibration and validation samples (Figure 3). The ratios of the standard error of prediction to standard deviation (RPD) for calibration and validation samples were 2.09 and 2.37, respectively, thus showing poor prediction accuracy. The number of PLS components for the final calibration model was 10.

### 3.3. Total Phenols

The level of the measured total phenol content in the sample was 34.68 ± 8.67 mg GAE 100 g^−1^, ranging from 16.77 to 60.91 mg GAE 100 g^−1^. The values of skewness and kurtosis were 0.68 and 3.06, respectively, indicating a positive skew in the sample.

**Figure 4:**
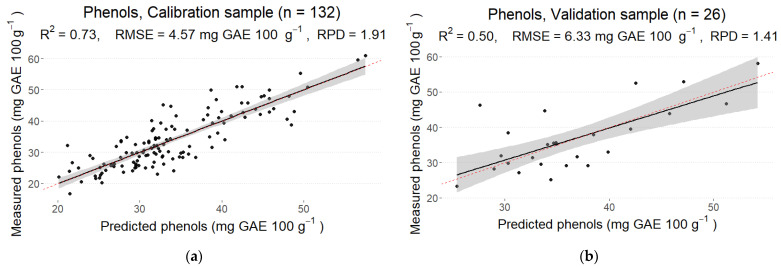
Predicted and measured values of phenols in the calibration (**a**) and prediction (**b**) sets with partial least squares regression (PLS) models.

The determination coefficient for the calibration sample was $R^{2}$ = 0.73 with RMSE = 4.57 mg GAE 100 g^−1^, indicating a somewhat good fit; however, $R^{2}$ = 0.50 with RMSE = 6.33 mg GAE 100 g^−1^ was obtained when applied to the validation sample, showing a poor fit for samples unseen in the calibration process (Figure 4). The ratios of the standard error of prediction to standard deviation (RPD) for calibration and validation samples were 1.91 and 1.41, respectively, thus showing a very poor prediction accuracy. The number of PLS components was 21.

### 3.4. Flavonoids

The level of measured flavonoid content in the sample was 4.73 ± 2.04 mg QE 100 g^−1^, and it varied from 1.09 to 11.02 mg QE 100 g^−1^. The sample skewness and kurtosis were 0.38 and 2.98, respectively, indicating a positive skewness with some relatively large sample values; however, not necessarily considered outliers.

**Figure 5:**
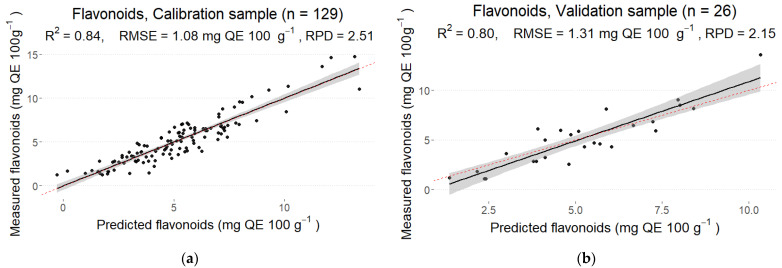
Predicted and measured values of flavonoids in the calibration (**a**) and prediction (**b**) sets with PLS models.

The determination coefficient for the calibration sample was $R^{2}$ = 0.84 with RMSE = 1.08 mg QE 100 g^−1^ while $R^{2}$ = 0.80 with RMSE = 1.31 mg QE 100 g^−1^ was obtained for the validation sample, indicating a good fit for both calibration and validation samples (Figure 5). The ratios of the standard error of prediction to standard deviation (RPD) for calibration and validation samples were 2.51 and 2.15, respectively, indicating a fair prediction accuracy. The optimal number of PLS components in the final model was 21.

### 3.5. Taste Index

The taste index is a dimensionless quantity and the measured level of this index in the sample was 1.15 ± 0.16 with a total range between 0.86 and 1.52. The sample skewness and kurtosis were 0.37 and 2.24, respectively, indicating a positive skewness in the sample; however, no outliers were detected.

**Figure 6:**
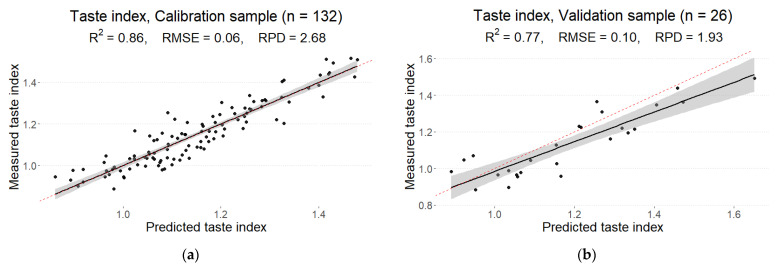
Measured and predicted values of taste index in the calibration (**a**) and prediction (**b**) sets derived from the best partial least squares regression (PLS) model from the reflectance spectrum with MSC pre-processing.

The final calibration model with RMSE = 0.06 resulted in $R^{2}$ = 0.86. The determination coefficient for the validation sample was $R^{2}$ = 0.77 with RMSE = 0.10, showing a worse but still acceptable fit compared to the calibration sample (Figure 6). According to the RPD metric, a fair model (RPD = 2.68) was obtained for the calibration sample, but a poor prediction model (RPD = 1.93) was obtained for the validation sample. The optimal number of PLS components was 25—the largest of all the models considered in this study.

### 3.6. Dry Matter

The level of measured dry matter content in the sample was 7.12 ± 1.82%, and it varied in the range between 4.55% and 13.15%. The sample skewness and kurtosis were 0.88 and 3.07, respectively, showing a positive skewness in the sample. Some more extreme values were observed compared to the rest of the sample; hence, care must be taken so that the validation sample does not contain them all.

**Figure 7:**
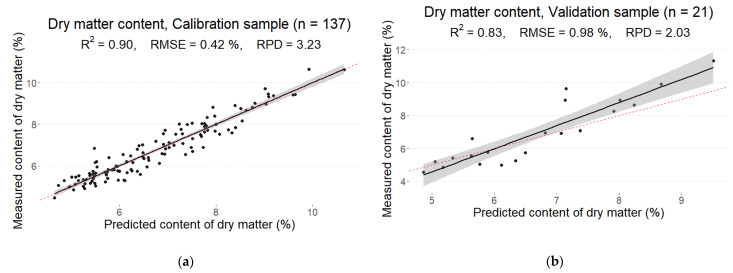
Measured and predicted values of dry matter in the calibration (**a**) and prediction (**b**) sets derived from the best partial least squares regression (PLS) model from the reflectance spectrum with MSC pre-processing.

The final calibration model with RMSE = 0.42% showed $R^{2}$ = 0.90, while the determination coefficient for the validation sample was $R^{2}$ = 0.83 with RMSE = 0.98%. Thus, a good performance was observed for both calibration and validation samples. According to the ratios of the standard error of prediction to standard deviation (RPD) metric, a good model (RPD = 3.23) was obtained for the calibration sample, but a poor prediction model (RPD = 2.03) was obtained for the validation sample. The optimal number of PLS components in the final model was 24 (Figure 7).

## 4. Discussion

Visible and near-infrared (Vis-NIR) spectroscopy is one of the methods used to identify various compounds in fruits and estimate their quality in a non-destructive manner [23,36]. The possibility of determining tomato quality attributes using Vis-NIR spectroscopy has been investigated in various studies [23,33,37,38]. Although this method can be applied in various sensing modes, the reflectance technique is one of the best ways to determine internal fruit quality attributes [39]. In our study, intact tomato fruits’ Vis-NIR reflectance spectra were used to develop partial least squares regression (PLS) models and predict the taste index, as well as the content of lycopene, flavonoids, β-carotene, dry matter, and total phenols. 

In the present study, the linear regression model of lycopene ($R^{2}$ = 0.90) produced a better fit than the content of β-carotene ($R^{2}$ = 0.82). Similar results were reported by Tilahun et al. [40]. They obtained good lycopene determination accuracy in the calibration ($R^{2}$ = 0.89) and prediction ($R^{2}$= 0.85) sets; however, the content of β-carotene had lower prediction accuracy in both the calibration ($R^{2}$ = 0.88) and prediction ($R^{2}$ = 0.77) sets. Moreover, linear regression results for lycopene content obtained by Li et al. [41] also demonstrated similar performance to that observed in our study. They obtained a model with high accuracy using the genetic algorithm partial least square regression method (the correlation coefficient was 0.907, and the root mean squared errors of cross-validation and prediction were 8.76 and 8.93, respectively). 

Other similar studies showed poorer performance for the PLS linear regression of carotenes. Baranska et al. [42] reported lower prediction accuracy of lycopene and β-carotene using NIR spectroscopy (regression coefficient was 0.85 and 0.80, respectively). However, the study performed by Goisser et al. [27] showed more accurate performance for the PLS linear regression of lycopene content. They used three portable NIR spectrometers to predict lycopene content in the intact tomato ‘Avalantino’. A good linear fit was obtained using an F-750 Produce Quality meter (Portland, OH, USA) followed by H-100F (Incheon, Republic of Korea) (coefficient of determination was 0.96 and 0.95, respectively). For CiO™ (Hod HaSharon, Israel), good performance was observed, and the coefficient of determination was 0.92. In the present study, fruits were tested only at the full ripeness stage. However, all ripening stages (green, breaker, turning, light red, and red) tested by Goisser et al. [27] resulted in better prediction capabilities, indicating the importance of data variation for the accurate prediction of lycopene. 

In our study, good flavonoid content determination accuracy was observed in calibration ($R^{2}$ = 0.84) and validation sets ($R^{2}$ = 0.80); however, the content of total phenols had lower prediction accuracy in both calibration ($R^{2}$ = 0.73) and validation ($R^{2}$ = 0.50) sets. A superior prediction performance was obtained in other studies. Alenazi et al. [26] used a near infra-red (NIR) spectrometer (F-750, Produce Quality Mater, Felix Instruments, Camas, WA, USA) at a wavelength range of 285–1200 nm to evaluate the tomato ‘Red Rose’ quality traits. They reported a higher coefficient of determination for total phenolic content in calibration ($R^{2}$ = 0.966) and validation sets ($R^{2}$ = 0.834). Moreover, higher prediction accuracy was observed for total phenolic content in calibration ($R^{2}$ = 0.984) and validation sets ($R^{2}$ = 0.790). In their study, five different ripening stages of tomatoes were used for the analysis, indicating the importance of data variation to obtain more precise linear regression models. 

Applying linear regression to quantify dry matter content resulted in a fit of $R^{2}$= 0.90 and $R^{2}$ = 0.83 for calibration and validation sets, respectively. Similar results were obtained in previous studies. Radzevičius et al. [43] used a NIR Case NCS001A (Sacmi Imola S.C., Bologna, Italy) spectrometer at a wavelength range of 600–1000 nm to evaluate the main quality attributes in tomatoes. They observed a high correlation for dry matter (regression coefficient was 0.91), indicating good prediction accuracy. Acharya et al. [44] reported high prediction accuracy of dry matter using a portable “Nirvana” SWNIR spectrometer (the coefficient of determination for the prediction set ranged from 0.86 to 0.92). In comparison to our study, the poorer prediction accuracy of dry matter content was obtained by Arruda de Brito et al. [33]. They used a portable Vis-NIR spectrometer (Felix Instruments, model F-750, Camas, WA, USA) and reported a coefficient of determination for dry matter prediction of $R^{2}$ = 0.62 and 0.59 for calibration and validation sets, respectively. 

In the presented study, a good prediction model was obtained for the taste index ($R^{2}$ = 0.86 and 0.77 for the calibration and validation sets, respectively). This index is calculated based on soluble solids content (SSC) and titratable acidity (TA), as indicated by Soare et al. [29]. Oliveira et al. [32] used a multi-purpose analyzer (MPA) spectrometer (Bruker Optics) in a reflectance mode to determine internal sensory attributes in various fruit species, including tomatoes. Excellent results were obtained for the apricot, and the regression fit of ($R^{2}$ = 0.95 and 0.93) was obtained for TA and SSC, respectively. In contrast, the notably poorer fit of ($R^{2}$ = 0.51 and 0.52) was observed for TA and SSC in tomatoes, respectively. Such differences were mainly attributed to the structural differences between the fruits. A superior regression fit for taste-related compounds was obtained in the study by Najjar and Abu-Khalaf [25]. They used Vis-NIR spectroscopy with a USB 2000+ miniature fiber optic spectrometer (Ocean Optics, Orlando, FL, USA) at a wavelength range of 550–1100 nm to model quality parameters in three varieties of tomato (i.e., ‘Ekram’, ‘Harver’, and ‘Izmer’) at a light red stage. A good prediction of soluble solids content (SSC), titratable acidity (TA), and taste (SSC/TA) was obtained (the coefficient of determination for the validation set was 0.98, 0.89, and 0.94, respectively). 

Nevertheless, obtained results in our study are satisfactory since the error range in predicting the taste index’s reference values was lower than 1% (root mean squared error of calibration and validation was RMSE = 0.05% and 0.10%, respectively). Furthermore, 80 varieties that vary in color, shape, size, and internal structure were used, which could affect prediction performance as indicated by previous studies. Research performed by de Oliveira et al. [32] proved that internal fruit heterogeneity is one of the major limiting factors for the precise prediction of taste-related compounds in tomatoes. Ibáñez et al. [45] reported that calibrations are necessary for each assay to obtain NIR spectra partial least square regression (PLS) models with a precise prediction of tomato taste-related compounds. Borba et al. [24] reported that separated calibration models are necessary for large and small varieties of tomatoes to obtain models with a higher prediction performance of soluble solids content and titratable acidity. 

According to Williams et al. [46], the determination coefficient of $R^{2}$ = 0.92 or higher indicates that the obtained model is suitable for fruit quality assessment; however, lower values ($R^{2}$ = 0.83 to 0.90) indicate that the obtained model can be used with caution for most applications, including research. Moreover, the ratios of the standard error of prediction (SEP) to standard deviation (SD) or RPD values must be higher than three to use the models for quality control [46]. Therefore, in our study, good results were obtained for lycopene and dry matter content, and the ratios of the standard error of prediction to standard deviation were RPD = 3.23 and RPD = 3.16, respectively. However, there is a need for further research to increase the robustness of the obtained models for the taste index, flavonoids, β-carotene, and total phenols for the application in fruit quality control. 

## 5. Conclusions

The results of the present study confirmed that visible and near-infrared (Vis-NIR) spectroscopy is a precise non-destructive method to determine tomato fruit quality in a single scan. The portable Vis-NIR spectroradiometer RS-3500 (Spectral Evolution Inc., Haverhill, MA, USA) allowed the quick and simultaneous quantification of multiple internal quality traits of the tomato. The models suitable to predict the taste index and the lycopene, flavonoid, β-carotene, total phenol, and dry matter content were developed using the partial least squares regression (PLS) algorithm. Although 80 varieties of tomato were used that have large differences in fruit size, shape, color, and internal structure, a strong correlation was observed between reflectance spectra data and the reference values obtained using a biochemical analysis for lycopene and dry matter. The results of the present study are in-line with previous research, indicating a high potential for non-destructive measurements in fruit quality control. Fast and cost-effective measurements are essential to ensure the high quality of fruits desired by breeders, producers, and consumers. Therefore, further research is required to increase the robustness of the current models for the prediction of a taste index, flavonoids, β-carotene, and total phenols.

## Figures and Tables

**Table 1:** Methods used to determine tomatoes’ quality parameters.

Parameters	Methods of Analyses	Reference
Lycopene	Solvent—tetrahydrofuran, spectrophotometry	[28]
β-carotene	Solvent—tetrahydrofuran, spectrophotometry	[28]
Total phenols	Solvent—methanol, spectrophotometry	[30]
Total flavonoids	Solvent—ethanol, spectrophotometry	[31]
Dry matter	Gravimetry	[28]
Soluble solids	Refractometry	[28]
Titratable acidity	Alkaline titration	[28]
Taste index	Calculations using soluble solids and titratable acidity	[29]

## Data Availability

The data presented in this study are available on request from the corresponding author.
